# 735. Vancomycin-Resistant *vanB*- and *vanA/vanB*-type *Enterococcus faecium* Causing Invasive Infections in Adult Patients in Chile

**DOI:** 10.1093/ofid/ofad500.796

**Published:** 2023-11-27

**Authors:** Lorena Diaz, Lina M Rivas, Jose R W Martinez, Anne S Peters, Maria Paz Riquelme, Rodrigo de Paula Baptista, Diana Panesso, Truc T Tran, Kavindra Singh, William R Miller, Yohanna Antolinez, Valentina Sanfurgo, Francisca Bugueno, Valeria E Quiroz, Katherine D Soto, Ana M Quesille-Villalobos, Pamela Rojas, Maria Rioseco, Juan Moreno, Araos Rafael, Cesar A Arias, José M Munita, Patricia Garcia

**Affiliations:** Universidad Del Desarrollo, Santiago, Region Metropolitana, Chile; Universidad del Desarrollo, Santiago, Region Metropolitana, Chile; Universidad del Desarrollo, Santiago, Region Metropolitana, Chile; Universidad del Desarrollo, Santiago, Region Metropolitana, Chile; Universidad del Desarrollo, Santiago, Region Metropolitana, Chile; Houston Methodist Hospital, Houston, Texas; Houston Methodist Research Institute, Houston, Texas; Houston Methodist Hospital, Houston, Texas; Houston methodist research institute, Houston, Texas; Houston Methodist Research Institute, Houston, Texas; Universidad del Desarrollo, Santiago, Region Metropolitana, Chile; Universidad del Desarrollo, Santiago, Region Metropolitana, Chile; Universidad del Desarrollo, Santiago, Region Metropolitana, Chile; Universidad del Desarrollo, Santiago, Region Metropolitana, Chile; Universidad del Desarrollo, Santiago, Region Metropolitana, Chile; Universidad del Desarrollo, Santiago, Region Metropolitana, Chile; Hospital Padre Hurtado, Santiago, Region Metropolitana, Chile; Hospital de Puerto Montt, Puerto Montt, Region Metropolitana, Chile; Hospital de Iquique, Iquique, Region Metropolitana, Chile; Universidad del Desarrollo, Santiago, Region Metropolitana, Chile; Houston Methodist and Weill Cornell Medical College, Houston, TX; Clínica Alemana - Universidad del Desarrollo, Santiago, Chile; Pontificia Universidad Católica de Chile, Santiago, Region Metropolitana, Chile

## Abstract

**Background:**

Vancomycin-resistant enterococci has increased globally in recent decades, with *Enterococcus faecium* (VR*Efm*) being the most prevalent species. Although the prevalence of VR*Efm* in Chile is high (∼70%), its molecular epidemiology is not well-described. Here, we used whole genome sequencing (WGS) to characterize the circulating lineages of VR*Efm* in Chile.

**Figure:**
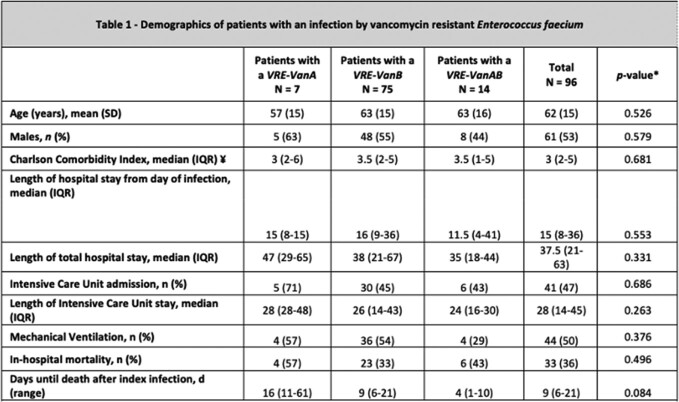

VRE= Vancomycin resistant Enterococcus faecium, SD=Standard deviation, IQR=in˝ter-quartile range; ¥ comorbidities included diagnoses apart from the primary cause for admission such as hypertension, chronic kidney disease and coronary artery disease; *Chi-square or Fisher´s exact were used for categorial variables; T-test for continues variables; P value<0.05 was considered significant.•

**Methods:**

A total of 96 invasive clinical VR*Efm* isolates causing invasive infections were collected from 96 patients admitted to 3 hospitals in Chile as part of an ongoing surveillance program (2018 – 2022). Clinical data including demographics, source of infection, and hospital outcomes were collected. All isolates were subjected to WGS on an Illumina platform. After assembly, *in silico* MLST and resistome were determined. Further, 5 representative strains were sequenced with Oxford Nanopore Technology (ONT) to further characterize the location of the *van* gene cluster.

**Results:**

The mean age of the cohort was 62 years, with 61% of males and a mean Charlson Comorbidity Score of 3. All isolates were resistant to vancomycin, ampicillin and ciprofloxacin and remained susceptible to linezolid. Resistance to teicoplanin (16%), high-level resistance to gentamicin (48%) and streptomycin (20%) was also observed. A total of 75 VR*Efm* harbored *vanB,* with the most common STs being ST17 (23%) and ST656 (25%). Only 7 VREfm isolates carried *vanA*, 43% of which corresponded to ST17. Surprisingly, 14 VR*Efm* simultaneously carried *vanA* and *vanB* (*vanA/vanB*), most of them belonging to ST233 (50%) or ST2137 (36%). For the VR*Efm vanA/vanB* isolates, the *vanB* cluster was located on the chromosome, while the *vanA* gene cluster was harbored on a plasmid (ca. 45Mb) which also contained *ermB*. A summary of the clinical characteristics of the cohort is shown in Table 1.

**Conclusion:**

*vanB* was the most frequently observed genotype in Chile, with ST17 and ST656 accounting for most isolates. Importantly, a substantial proportion of VR*Efm* carried both *vanA* and *vanB* clusters in Chile, which was mainly observed in two different genomic lineages (ST233 and ST2137). Active surveillance of VR*Efm* is essential to monitor the epidemiology of this critical pathogen in South America.

**Disclosures:**

**William R. Miller, M.D.**, Merck: Grant/Research Support|UpToDate: Honoraria

